# A unified approach to protein domain parsing with inter-residue distance matrix

**DOI:** 10.1093/bioinformatics/btad070

**Published:** 2023-02-03

**Authors:** Kun Zhu, Hong Su, Zhenling Peng, Jianyi Yang

**Affiliations:** School of Mathematical Sciences, Nankai University, Tianjin 300071, China; School of Mathematical Sciences, Nankai University, Tianjin 300071, China; Ministry of Education Frontiers Science Center for Nonlinear Expectations, Research Center for Mathematics and Interdisciplinary Sciences, Shandong University, Qingdao 266237, China; Ministry of Education Frontiers Science Center for Nonlinear Expectations, Research Center for Mathematics and Interdisciplinary Sciences, Shandong University, Qingdao 266237, China

## Abstract

**Motivation:**

It is fundamental to cut multi-domain proteins into individual domains, for precise domain-based structural and functional studies. In the past, sequence-based and structure-based domain parsing was carried out independently with different methodologies. The recent progress in deep learning-based protein structure prediction provides the opportunity to unify sequence-based and structure-based domain parsing.

**Results:**

Based on the inter-residue distance matrix, which can be either derived from the input structure or predicted by trRosettaX, we can decode the domain boundaries under a unified framework. We name the proposed method UniDoc. The principle of UniDoc is based on the well-accepted physical concept of maximizing intra-domain interaction while minimizing inter-domain interaction. Comprehensive tests on five benchmark datasets indicate that UniDoc outperforms other state-of-the-art methods in terms of both accuracy and speed, for both sequence-based and structure-based domain parsing. The major contribution of UniDoc is providing a unified framework for structure-based and sequence-based domain parsing. We hope that UniDoc would be a convenient tool for protein domain analysis.

**Availability and implementation:**

https://yanglab.nankai.edu.cn/UniDoc/.

**Supplementary information:**

Supplementary data are available at *Bioinformatics* online.

## 1 Introduction

The protein domain is defined as the basic unit of proteins, which can fold and carry out biological functions independently (Wetlaufer, 1973). Decomposition of multi-domain proteins into domains is fundamental for precise domain-based structure prediction and functional characterizations. To decompose the protein structures in the Protein Data Bank (PDB) (Berman *et al.*, 2000), a few structure-based domain databases were developed, including SCOP (Murzin *et al.*, 1995), CATH (Orengo *et al.*, 1997) and ECOD (Cheng *et al.*, 2014). It is non-trivial to decompose protein structures into domains, especially when the boundaries are blurred (Postic *et al.*, 2017). For example, manual annotations are conducted in the construction of the SCOP databases.

A few automated algorithms were developed to decompose protein structures into domains. PDP (Alexandrov and Shindyalov, 2003) is one of the widely used programs that maximize intra-domain interactions while minimizing inter-domain interactions. DomainParser (Guo *et al.*, 2003) uses a graph-theoretic approach to formulate the domain decomposition problem as a network flow problem. DDOMAIN (Zhou *et al.*, 2007) divides protein into domains using a normalized contact-based domain–domain interaction profile. SWORD (Postic *et al.*, 2017) aims at cutting protein structure into alternative domains, using a hierarchical clustering procedure to combine the protein units. However, the application of this method can be limited by its slower speed. These methods tend to overcut protein domains and the original secondary structure may be destroyed after domain decomposition. Eguchi and Huang (2020) trained a convolutional neural network model for performing semantic segmentation of protein structures. Although this method is designed to solve a classification problem that is distinct from domain parsing, the architecture can be adapted to perform domain parsing with comparable performance to, e.g. SWORD and PDP.

As many proteins do not have experimental structures, another closely related research is the sequence-based prediction of the domain boundaries. Many methods have been developed for this purpose, including ThreaDom (Xue *et al.*, 2013), DOMPro (Cheng *et al.*, 2006), ConDo (Hong *et al.*, 2019), and DNN-dom (Shi *et al.*, 2019), CHOP (Liu and Rost, 2004), FIEFDOM (Bondugula *et al.*, 2009), FUpred (Zheng *et al.*, 2020), etc. ThreaDom, CHOP and FIEFDOM are template-based approaches, which infer the domain boundaries by identifying homologous templates with known domain information. However, this type of method does not perform well when the sequence similarity is not sufficient (e.g. <30%). DOMPro, ConDo and DNN-dom are machine-learning-based methods. They take the input of coevolutionary information, the predicted secondary structure, the predicted solvent accessibility and the sequence profile to train a machine-learning model to predict domain boundaries. Their predictive accuracy is in general lower than the template-based methods. FUpred is a contact-based approach, which detects domain boundaries from predicted contact map. Its accuracy of the domain parsing depends on the accuracy of the predicted contact map.

With the advance in deep learning-based structure prediction methods (Jumper *et al.*, 2021; Su *et al.*, 2021), it becomes much more accurate and faster than before to predict protein structure from protein sequence. However, it remains hard to predict accurate structure for big proteins (e.g. >3000 amino acids). In such case, we may still be able to predict relatively accurate inter-residue distances between domains, which is relatively easier to predict than protein structure. It is known that correct assignment of domains can lead to improved structure prediction for individual domains. Therefore, sequence-based domain parsing is also meaningful.

The advance in protein structure prediction provides the opportunity to deal with the problems of the sequence-based and the structure-based domain decompositions under a unified framework. In this work, we introduce a new domain parsing method UniDoc, which can predict more accurate domain boundaries using the inter-residue distance matrix that can be derived from the input structure or predicted by trRosettaX (Su *et al.*, 2021).

## 2 Materials and methods

### 2.1 Benchmark datasets

Five benchmark datasets (four for structure-based and one for sequence-based domain parsing) are used in this work, which are summarized in Table 1.

**Table 1.** Summary of the benchmark datasets

Dataset	#Single/Multi	Definition	Type
Islam90	68/19	M	3D
Broad-consensus	273/55	S+C+E+M	3D
Consensus	2841/682	S+C+E	3D
Weak-consensus	2231/538	S+C	3D
FUpred_seq	1700/849	S	1D

S, SCOP; C, CATH; E, ECOD; M, Islam2363.

Each dataset contains single-domain (denoted by Single) and multi-domain (denoted by Multi) proteins. For the multi-domain proteins, we give a more detailed classification (Supplementary Table S1) based on the number of domains and whether they are continuous. The first three structure-based datasets in the table are from the work of SWORD (Postic *et al.*, 2017). The main difference between these datasets is the domain definition from different databases (SCOP, CATH, ECOD and Islam2363). For example, the domains in the Broad-consensus data set have similar annotations in CATH, SCOP, ECOD and Islam2363 (Islam *et al.*, 1995), while the domains in the Consensus dataset have similar annotations in CATH, SCOP and ECOD. Since Broad-consensus has more strict domain definitions, it has fewer number of proteins than Consensus. Islam2363 (Islam *et al.*, 1995) is a domain dataset which contains 2363 manually annotated domain assignments. We also constructed a new structure-based non-redundant dataset (denoted by Weak-consensus) by considering consistent domain annotations in SCOP and CATH. It consists of 2769 proteins with pairwise sequence identify ≤30%. All proteins that are identical in the Consensus are removed in the Weak-Consensus, which results in a smaller amount of data. The last dataset (FUpred_seq) is from the work of FUpred (Zheng *et al.*, 2020), which is used to evaluate sequence-based domain recognition.

### 2.2 Domain interaction score

The division of a protein structure into domains is based on the intuition that the interaction inside the domains (i.e. intra-domain interaction) is stronger than that between domains (inter-domain interaction). To quantitively measure the domain interactions, we define domain interaction score (DIS) based on the inter-residue interaction as follows. It can be characterized by the *C_β_*–*C_β_* (*C_α_*–*C_α_* for Glycine) distance matrix *D*. The distance is transformed into contact probability between 0 and 1 using the following transform:

(1) $$p_{ij}=\frac{1}{1+e^{\left(d_{ij}-d_{0}\right)/\delta }},$$

where *d_ij_* is the distance between the *i*th residue and the *j*th residue. The values for *d_0_* and *δ* are 8.0 and 1.5 Å, respectively (Gelly *et al.*, 2006).

The inter-domain interaction score is defined as:

(2) $${\text{DIS}}_{\text{inter}}(D_{1},D_{2})=\frac{1}{l_{1}{}^{\alpha }l_{2}{}^{\alpha }}\sum_{i\in D_{1}}^{}\sum_{j\in D_{2}}^{}p_{ij},$$

where *D*_1_ and *D*_2_ are two domains with sizes *l*_1_ and *l*_2_, respectively; *p_ij_* is defined by Equation (1). The parameter *α* is 0.43 according to Alexandrov and Shindyalov (2003).

The intra-domain interaction score is defined as:

(3) $${\text{DIS}}_{\text{intra}}(D)=\frac{1}{l^{\beta }}\sum_{i\in D}^{}\sum_{j\in D,|i-j|>2}^{}p_{ij},$$

where *D* is a domain of *l* amino acids; the parameter *β* is 0.95 empirically. In Supplementary Figure S1, an example is given to visualize both scores.

### 2.3 Framework of UniDoc

**Fig. 1.**
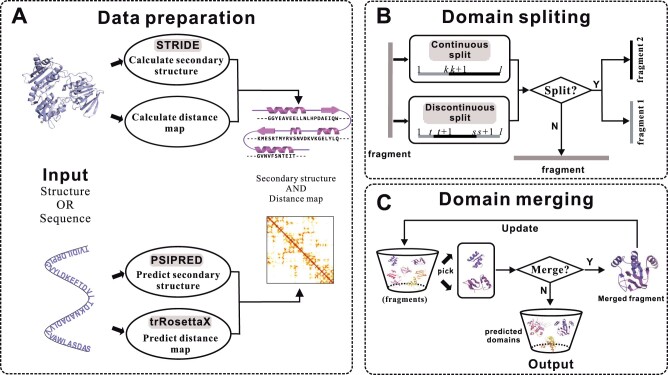
Framework of UniDoc. (**A**) The input to UniDoc can be either a structure or a protein sequence. (**B**) Top-down algorithm to decompose a fragment into two sub-fragments using either a continuous or discontinuous split. (**C**) Bottom-up algorithm for merging fragments

The overall architecture of UniDoc is shown in Figure 1. Its idea is similar to the structure-based approach PDP. The input can be either a 3D structure or a protein sequence (Fig. 1A). When the 3D structure is available, the inter-residue distance map is calculated and the secondary structure is defined by STRIDE (Heinig and Frishman, 2004). When protein sequence is provided, trRosettaX (Su *et al.*, 2021) and PSIPRED (McGuffin *et al.*, 2000) are applied to predict the distance map and the secondary structure, respectively.

With the distance map and the secondary structure, a two-step approach is used to predict domain boundaries. The first step splits a protein into fragments (‘top-down’, Fig. 1B) while the second step merges the fragments (‘bottom-up’, Figure 1C) to obtain the final domain decomposition. More details are presented below.

### 2.4 Top-down algorithm for splitting

The principle of splitting a protein into domains is to minimize the interaction between domains. Two constraints are required in the procedure of split. The first one is the size of a fragment should be longer than 30. The second one is a cutting position should not be from regular secondary structures (*α*-helix and *β*-sheet). We use two approaches to decompose a protein into domains, including continuous and discontinuous splits (Fig. 1B). The one resulting in the smallest interaction score is selected at each step. At the beginning of the algorithm, the whole protein is regarded as a single-domain protein consisting of a continuous fragment.

**Continuous split.** Given a candidate fragment (*D*), a continuous split is defined as the split that results in two continuous sub-fragments (*D*_1_ and *D*_2_). A single cutting position is needed for such a split (Supplementary Fig. S2A). The optimal cutting position can be obtained by solving the following equation:

(4) $$k=\underset{1<k^{\prime }<l}{\mathrm{argmin}}\;{\text{DIS}}_{\text{inter}}\;(D_{1}^{k^{\prime }},D_{2}^{k^{\prime }}),$$

where *l* is the length of *D*; *D*_1_^*k'*^ and *D*_2_^*k'*^ are the resulting sub-fragments at the cutting position *k'*.

**Discontinuous split.** A discontinuous split is defined as the split that results in one discontinuous fragment (*D*_1_) and one continuous fragment (*D*_2_). Two cutting positions are needed for such a split (Supplementary Fig. S2B). The optimal cutting positions *t* and *s* can be obtained with the following equation:

(5) $$\left(t,s)=\underset{\begin{array}{c} 1<t^{\prime },s^{\prime }<l,\\ s^{\prime }>t^{\prime }+35,\\ d_{s^{\prime }t^{\prime }}<8Å \end{array}}{\mathrm{argmin}}{\;\text{DIS}}_{\text{inter}}(D_{1}^{t^{\prime },s^{\prime }},D_{2}^{t^{\prime },s^{\prime }}\right),$$

where *D*_1_^*t′, s′*^ and *D*_2_^*t′, s′*^ are discontinuous and continuous sub-fragments, respectively, from the cutting positions *t′* and *s′*. Note that the above equation implies that two key constraints are forced to ensure the splitting is meaningful. First, the two cutting points should not be too close to each other in the 1D sequence (>35) to ensure that the resulting continuous fragment is not too small. Second, the two cutting points should not be too far away from each other in the 3D structure to ensure that the sequentially distant sites are spatially close.

To partly address the issue of over-splitting (i.e. splitting more number of domains than the native definition), the interaction score between the sub-fragments after splitting is compared with the interaction score of the original fragment (DIS_intra_) as follows. First, we select the optimal split with the smallest interaction score (DIS_inter_) from the continuous and the discontinuous splits. Second, this split is accepted only when DIS_inter_ is less than half of DIS_intra_; otherwise, no split is performed on this fragment to avoid over-splitting.

Note that the top-down approach ensures that the interactions between the resulted sub-fragments from each step are minimized; however, it does not guarantee that the interactions among sub-fragments from multiple steps are minimized. This may be because the top-down approach only captures local interactions in each step. To solve this problem, a bottom-up algorithm is applied to merge sub-fragments that have strong interactions, as detailed below.

### 2.5 Bottom-up algorithm for merging

A bottom-up algorithm is applied iteratively to merge the fragments from the top-down splitting into domains to maximize the interactions inside each domain as follows (Fig. 1C). Two fragments (*D_I_* and *D_J_*) are selected from all fragment pairs to maximize the score defined by the following formula:

(6) $$S(i,j)={\text{DIS}}_{\text{inter}}(D_{i},D_{j})-\min\{{\text{DIS}}_{\text{intra}}(D_{i}),{\text{DIS}}_{\text{intra}}(D_{j})\},$$

where *i* and *j* are between 1 and the total number of fragments from the top-down splitting. The fragments *D_I_* and *D_J_* are merged, if *S*(*I*, *J*) is positive, meaning that the interaction between these fragments is stronger than within one of the individual fragments; otherwise, the merging step is done.

A post-processing step is further applied to merge less compact fragments. A fragment with weak intra-domain contact (DIS_intra_ <1.0) will be merged with another fragment that has the strongest interaction with it (i.e. the one with the highest value of DIS_inter_).

### 2.6 Performance evaluation

The performance of classifying single and multi-domain proteins is measured mainly based on the metric Matthew’s correlation coefficient (MCC). In addition, the precision and the accuracy are also calculated.

For structure-based domain parsing, the metric CDO (Correct Domain Overlap) score is used to measure the performance of a method (Alexandrov and Shindyalov, 2003; Postic *et al.*, 2017). CDO score is calculated as the ratio of targets with correct domain assignment (Supplementary Table S2). A domain assignment is considered correct if the following conditions are satisfied: (i) the predicted number of domains is consistent with the number of annotated domains; (ii) the overlap between each predicted domain and the reference is greater than a given threshold (denoted by *t*). Previously, this threshold was simply set to 85% (Alexandrov and Shindyalov, 2003; Postic *et al.*, 2017). We will discuss the impact of this threshold later.

For sequence-based domain boundary prediction, besides the above metrics, NDO (Normalized Domain Overlap) score (Tai *et al.*, 2005) is used (Shi *et al.*, 2019; Xue *et al.*, 2013; Zheng *et al.*, 2020). More details about the calculation of the above metrics are available in Supplementary Table S3.

## 3 Results and discussions

### 3.1 Structure-based domain parsing

#### 3.1.1 Classification of single- and multi-domain proteins

**Fig. 2.**
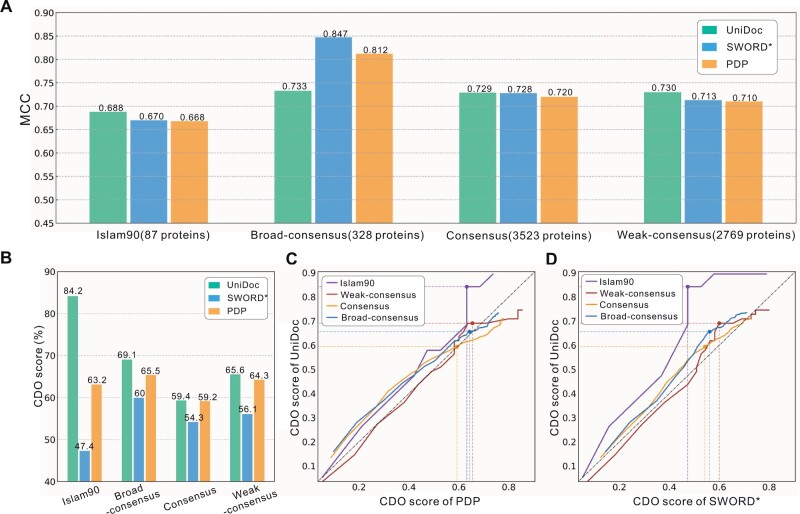
Performance for structure-based domain parsing. (**A**) The MCCs for UniDoc and the other two controlled methods (SWORD* and PDP) on four structure datasets. (**B**) The performance on multi-domain proteins in terms of CDO score at the domain overlap threshold of 85%. (**C**, **D**) are the CDO scores at varying overlap thresholds between 0 and 1. The dots represent the scores at the threshold of 85%

Based on the domain parsing result, we can infer if it is a single-domain or a multi-domain target. Figure 2A shows the MCCs for UniDoc and the other two controlled methods (SWORD* and PDP) on four structure datasets. Note that SWORD* refers to taking the default first-ranked prediction from SWORD. Except for the dataset Broad-consensus, UniDoc has a higher MCC than other methods on the other three datasets. The MCC may be easily affected by the wrong predictions for just a few multi-domain targets, given the small size of multi-domain proteins. The correctly predicted numbers of multi-domain proteins are 44, 48 and 51 for UniDoc, SWORD* and PDP, respectively. PDP (resp. SWORD*) has the highest recall at the expense of lower precision for multi-domain (resp. single-domain) targets, indicating that it tends to overcut (resp. undercut) the structures into domains (Supplementary Table S4).

#### 3.1.2 Performance on multi-domain proteins

Figure 2B–D summarizes the comparisons among UniDoc, SWORD* and PDP on multi-domain proteins (Supplementary Fig. S3 for proteins with >2 domains). Figure 2B suggests that when the domain overlap threshold is set to 85%, UniDoc outperforms the two controlled methods on all datasets. We also investigate the impact of this threshold (*t*). When this threshold is changed between 0 and 1, we calculate the corresponding CDO score on each dataset for each method. A higher/lower value of *t* means a more/less stringent requirement for an assignment to be regarded as correct, which thus results in a lower/higher CDO score. The results are shown in Figure 2C and D, for comparison with PDP and SWORD*, respectively. They suggest except for the small dataset Broad-consensus (55 targets), UniDoc has consistently higher CDO scores than both methods when the threshold becomes more stringent than 85%. This suggests that the domain assignments by UniDoc are in a higher resolution than the controlled methods. On the Broad-consensus dataset, UniDoc is competitive with PDP and SWORD*, as shown by the close distance between the diagonal line and the brown curve.

#### 3.1.3 Impact of ambiguous definition of domain boundary

When the domain boundary for a protein is blurred, the domain annotations by CATH and SCOP are not always consistent. CATH tends to cut proteins into smaller domains; while SCOP uses evolutionary and structural relationships to define domains, making the SCOP domains larger. This inconsistent domain definition may affect the performance assessment. A few case studies are given to illustrate this issue.

For the protein *APO-liver alcohol dehydrogenase* (PDB ID: 8ADH, chain A, Supplementary Fig. S4A), both SCOP and CATH decompose it as two domains but with different domain boundaries. CATH divides the *α*-helix (residues 165–188, highlighted in a circle in Supplementary Fig. S4A) into two segments; while SCOP keeps this *α*-helix as a whole. The domain predictions by PDP and SWORD* are consistent with the CATH definition, while UniDoc’s prediction is consistent with the SCOP definition. Meanwhile, the SWORD predictions are not consistent with the SCOP definition.

For the protein *X-ray crystal structures of trypsin complexes and thrombin complexes* (PDB ID: 1PHH, chain A, Supplementary Fig. S4B), it is difficult to distinguish the number of domains and their boundaries from the distance map. The boundary division for this example is very ambiguous. Although both CATH and SCOP divide this structure into two domains, the domain boundaries are different. Due to different assignments of the highlighted *β*-strands (residues 174–181 and residues 267–273) and the segments (residues 73–95 and 352–394), CATH assigns multiple discontinuous fragments in both domains; while SCOP keeps continuous fragments into one of the domains. The predictions by UniDoc and PDP are consistent with the SCOP definition while the SWORD* prediction is different from both CATH and SCOP definitions. None of the solutions by SWORD are similar to the definition by SCOP or CATH.

The last example is the protein *multifunctional methyltransferase* (PDB ID: 1PJQ, chain A, Supplementary Fig. S4C). From the distance map, we can see that there are sparse interactions among the residues 113–457. This makes the number of domains by CATH and SCOP different (5 and 3, respectively). The residues from 113 to 215 (highlighted in box) are divided into two domains by CATH and one by SCOP. The results of UniDoc and PDP are consistent with CATH; while the SWORD* prediction is consistent with SCOP. Similarly, CATH splits the residues from 216 to 457 (highlighted in box) into two domains while SCOP combines them in one domain. The predictions by all three methods are consistent with the CATH annotations. One of the solutions by SWORD is correct according to CATH; but it is not on the top in its default ranking.

#### 3.1.4 Running time analysis

We compare the time complexity of structure-based domain parsing by UniDoc, SWORD and PDP on the proteins from the dataset Broad-consensus. All three methods were run locally and the running time for all proteins was recorded. Supplementary Figure S5 shows that SWORD is much slower than UniDoc and PDP. On average, UniDoc takes 0.017 s to process one protein, which is more than two times faster than PDP (0.048 s per protein); and 312 times faster than SWORD (5.32 s per protein). The fast speed of UniDoc can be explained by the strategy employed, i.e. the cutting points that break the secondary structure are not considered. This decreases the time complexity while ensuring the precision of domain boundary prediction.

### 3.2. Sequence-based domain parsing

As the inter-residue distance prediction becomes much more accurate than before, we can apply the above structure-based domain parsing algorithm to the problem of sequence-based domain boundary prediction. The only difference is the inter-residue distance is predicted by trRosettaX rather than derived from the input structure.

Though many sequence-based domain boundary prediction methods are available, we only compare UniDoc with FUpred (Zheng *et al.*, 2020), which is the state-of-the-art method that uses predicted inter-residue contacts. FUpred was installed and executed locally. The comparison is based on the dataset from the FUpred work, which consists of 1700 single-domain proteins and 849 multi-domain proteins. We also derive the distance/contact from the native structure as inputs to UniDoc and FUpred, with the same definition of inter-residue contact in FUpred.

First, we compare the performances in classifying single-domain and multi-domain proteins by both methods. As shown in Supplementary Table S5, with the distance/contact map predicted by trRosettaX, the MCC of UniDoc is 0.804, which is slightly higher than FUpred (0.784). This is consistent with the reported MCC in the FUpred paper, i.e. 0.799 with predicted contact map ResPRE (Zheng *et al.*, 2020).

Second, the performances on parsing 849 multi-domain proteins by UniDoc and FUpred are compared based on CDO and NDO scores (Supplementary Table S5). Supplementary Figure S6 summarizes the results on proteins with >2 domains. The CDO score suggests that UniDoc correctly recognized the domain boundaries for 49.59% (=421/849) of multi-domain proteins, which is 4.24% higher than FUpred. This translates to a total of 36 proteins that are correctly predicted by UniDoc but not by FUpred. Note that for structure-based domain parsing (Fig. 2), the CDO score is above 60%, much higher than the data in Supplementary Table S5. This is likely because the domain definition for the proteins in this dataset is from SCOP only. Thus, it may inevitably have some proteins with ambiguous definitions of domain boundaries. To verify this hypothesis, for the multi-domain proteins in the FUpred-seq dataset, we compared the domain annotations in CATH and SCOP. It turns out that CATH and SCOP have different domain definitions for 458 proteins out of the 849 multi-domain proteins in this dataset.

Similar to the previous analysis, we compute the CDO scores at different overlap thresholds in Supplementary Figure S7A. It shows that UniDoc consistently outperforms FUpred at all thresholds. As the CDO score is binary, we further compare both methods based on the NDO score, which is widely used for measuring sequence-based domain recognition methods. Supplementary Table S5 shows that UniDoc achieves an NDO score of 0.812, slightly higher than FUpred (0.804). Note that FUpred’s NDO score was 0.791 with the predicted contact map by ResPRE, comparable with the data in Supplementary Table S5.

In addition, we compare the time complexity of UniDoc and FUpred on 849 multi-domain proteins in Supplementary Figure S7B. The time for input data preparation (including distance/map and secondary structure prediction) is not considered for both methods. On average, UniDoc takes 0.30 s per protein to predict domain boundaries, which is more than 2 times faster than FUpred (0.73 s per protein).

### 3.3 Impact of the accuracy of predicted distance

**Fig. 3.**
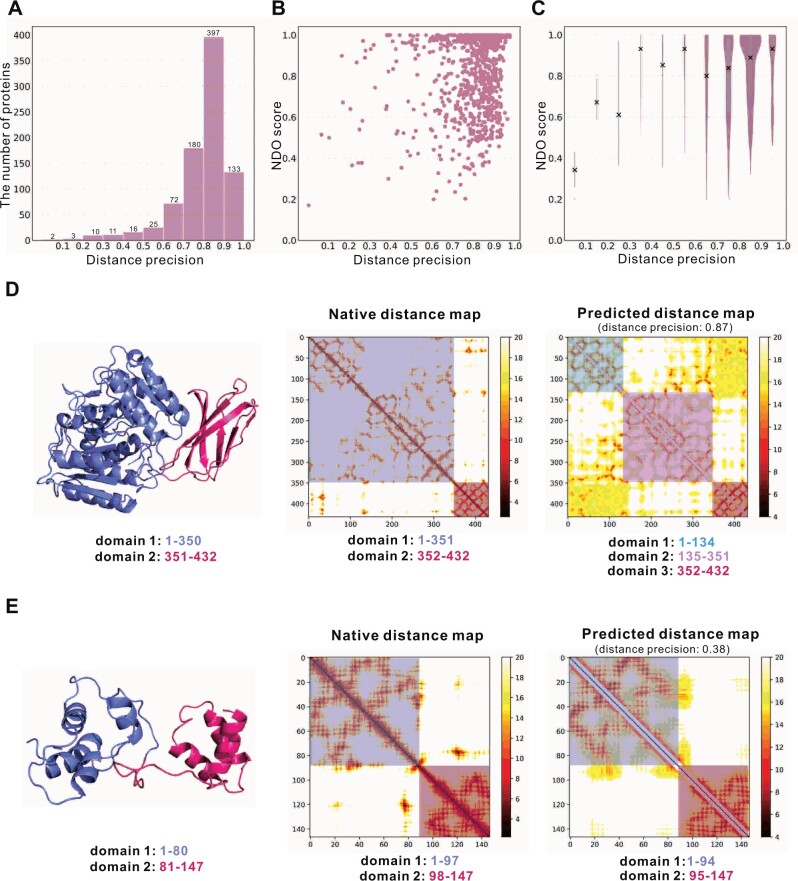
Correlation between the distance precision and the accuracy of domain parsing. (**A**) Distribution of distance precision on the multi-domain proteins from the dataset FUpred_seq. (**B**) Plot of the distance precision and the NDO score. (**C**) The violin figure between the distance precision and the NDO score (summarized from B). The distance precision is divided into 10 bins to calculate the distribution of the NDO scores. The mean scores are indicated by the cross signs. (**D, E**) Two examples to explain the low correlation between distance precision and the accuracy of domain boundaries prediction. The domain information under the cartoon structures is the ground truth; while those under the distance map are the predictions

Since UniDoc decomposes protein domains based on distance matrix, whether accurate distance prediction can be transferred into a correct prediction of domain boundaries. First, we compute the accuracy of the predicted distance [i.e. distance precision defined in Du *et al.* (2022)] for the 849 multi-domain proteins. Figure 3A shows that the distances for most (92%) proteins were predicted with a precision higher than 0.6, making it possible to infer correct domain boundaries from sequences. However, Figure 3B indicates that there is no clear correlation between the distance precision and the NDO score. This is probably because the distance precision measures the global accuracy while the NDO score measures the accuracy of boundary (kind of local measure) detection. Two representative examples are given in Figure 3D and E to illustrate this.

According to SCOP, the protein *crystal structure of the Arg-specific cysteine proteinase gingipain R* (PDB ID: 1CVR, chain A, Fig. 3D), has two domains (residues 1–350 and 351–432). Correct domain boundary was obtained when the native structure was used in UniDoc. The predicted distance for this protein is accurate with a distance precision of 0.87. However, the domain boundary was predicted wrongly from this distance (i.e. three domains) are incorrect with a CDO score of 0 and NDO score of 0.685. This is mostly because the inter-domain distance was predicted with poor precision though the intra-domain distance was predicted well.

The opposite of this example is the *solution structure of HNF-6* (PDB ID: 1S7E, chain A, Fig. 3E), which has two domains according to SCOP (residues 1–80 and residues 81–147). UniDoc made correct predictions with both the native and the predicted distances. Note that the predicted intra-domain distance is of low accuracy, resulting in a low global distance precision (0.383). However, the inter-domain distance was correctly predicted, making the domain boundary clear to infer.

We divide the distance precision from 0 to 1 into 10 bins and calculate the distribution of NDO scores in each bin. The numbers of proteins with distance precision in the range from 0 to 0.6 are very small (Fig. 3C), which has no statistical significance. When the distance precision is higher than 0.6, Figure 3C shows that the median value of the NDO score does become higher with a more accurate distance prediction.

## 4 Conclusions

Decomposition of multi-domain proteins into domains is of fundamental meaning for precise domain-based structure prediction and functional characterizations. The recent breakthroughs in deep learning-based protein structure prediction make it possible to unify structure-based and sequence-based domain parsing. We introduce one of such approaches, UniDoc, for unified domain parsing using either structure-derived or predicted inter-residue distance matrix. Comprehensive tests on five benchmark datasets show that UniDoc works very well, outperforming other peering methods, in terms of both accuracy and speed. Though with a high prediction accuracy, UniDoc still has a limit. Only one domain decomposition is given even if multiple alternative decompositions exist. More efforts are needed to deal with the proteins with ambiguous definition of domain boundaries. We anticipate that the release of UniDoc could contribute to the community for protein domain analysis.

## Supplementary Material

btad070_Supplementary_Data

## Data Availability

All data used in this work can be downloaded at: https://yanglab.nankai.edu.cn/UniDoc/benchmark/.
