# Supplementary material for: A unified approach to protein domain parsing with inter-residue distance matrix
Source: Bioinformatics. 2023 Feb 3;39(2):btad070. doi: 10.1093/bioinformatics/btad070 (PMC9919455; doi:10.1093/bioinformatics/btad070)
Supplement: btad070_Supplementary_Data [file btad070_supplementary_data.pdf]

Supplementary Materials

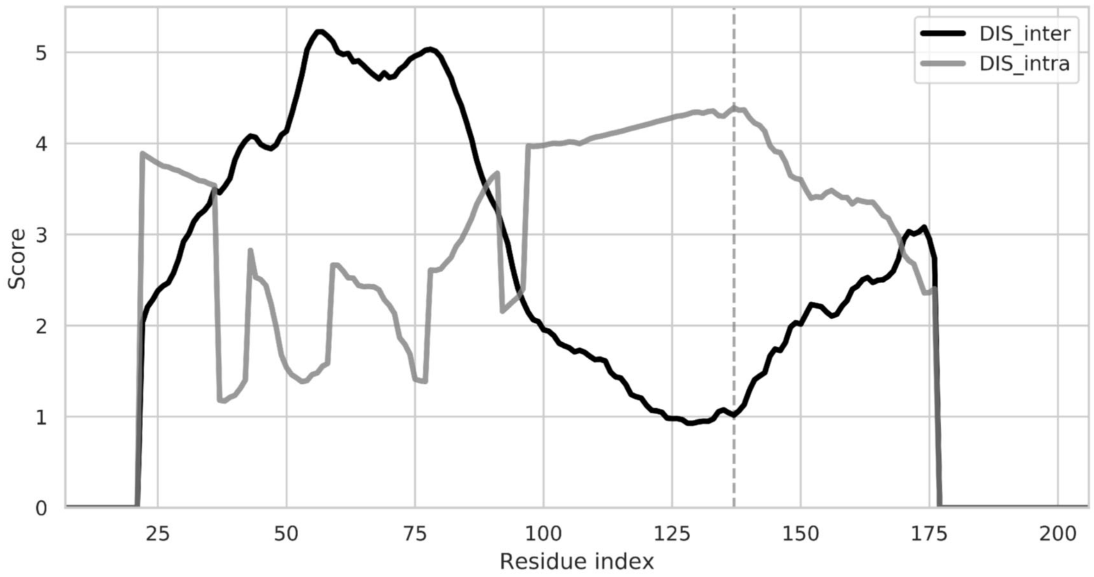

Fig. S1. An example (PDB ID: 1G6N, chain A) to visualize the change in intra-domain interaction scores and inter-domain interaction scores at different cutting points. The black line and gray line represent the distribution of DIS_inter and DIS_intra, respectively. The final split point (the dashed line) maximizes the intra-domain interaction and almost minimizes the inter-domain interaction. The final splitting point is not the exact minimum of the inter-domain interaction because the minimum is at the alpha-helix residue that is prohibited to be cut to keep the secondary structure.

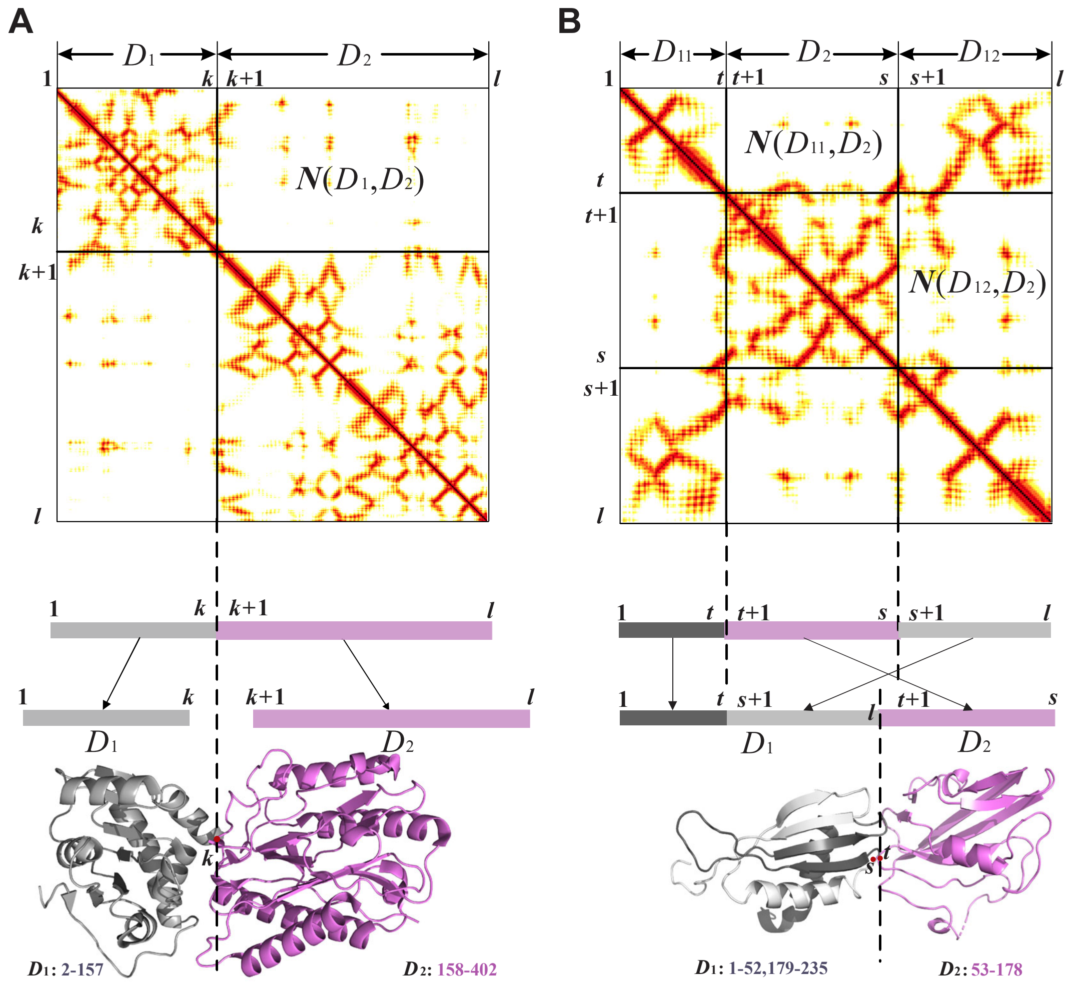

Fig. S2. Diagram of two approaches to decompose protein into domain segments. (A) The diagram of cutting a protein into two continuous domains. *k* is a candidate domain boundary. $N(D_1, D_2)$ in each block is defined as $N(D_1, D_2) = \sum_{i \in D_1, j \in D_2} p_{ij}$. (B) The diagram of decomposing a protein into a continuous domain and a discontinuous domain, *t* and *s* are the candidate domain boundaries.

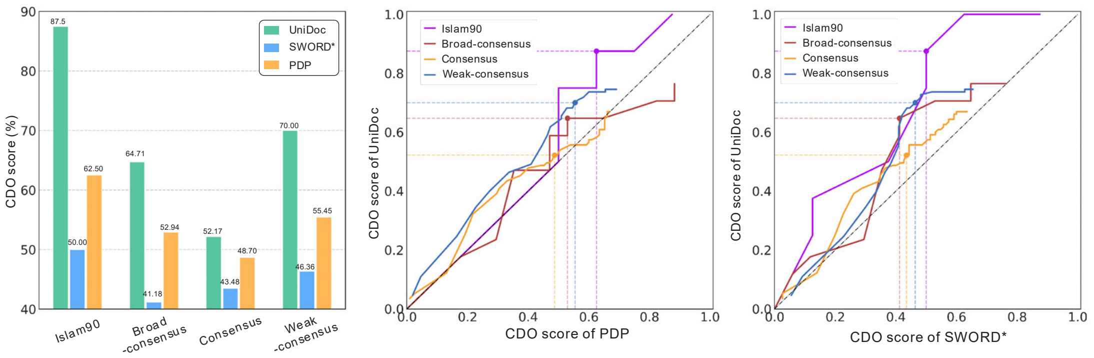

Fig. S3. Comparison with other structure-based methods on the subsets of structures consisting of >2 domains.

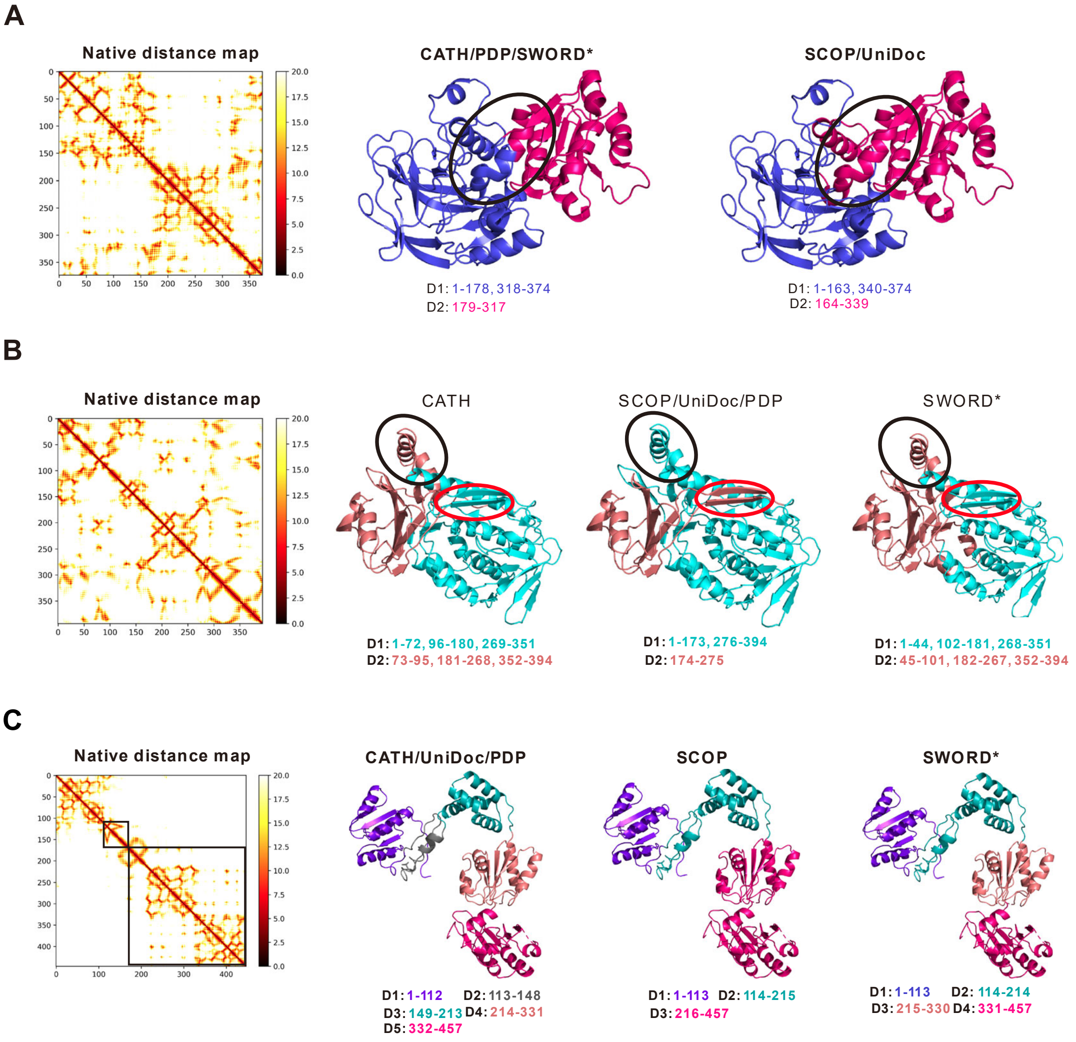

Fig. S4. Examples with the disputable definition of the domain boundary. These examples are from the dataset Islam90. (A) PDB ID: 8ADH, chain A. (B) PDB ID: 1PHH, chain A. (C) 1PJQ, chain A. The circles in (A, B) highlight the regions that have disputable domain definitions. The font colors for the numbers under each structure map to the colors in the cartoon structure.

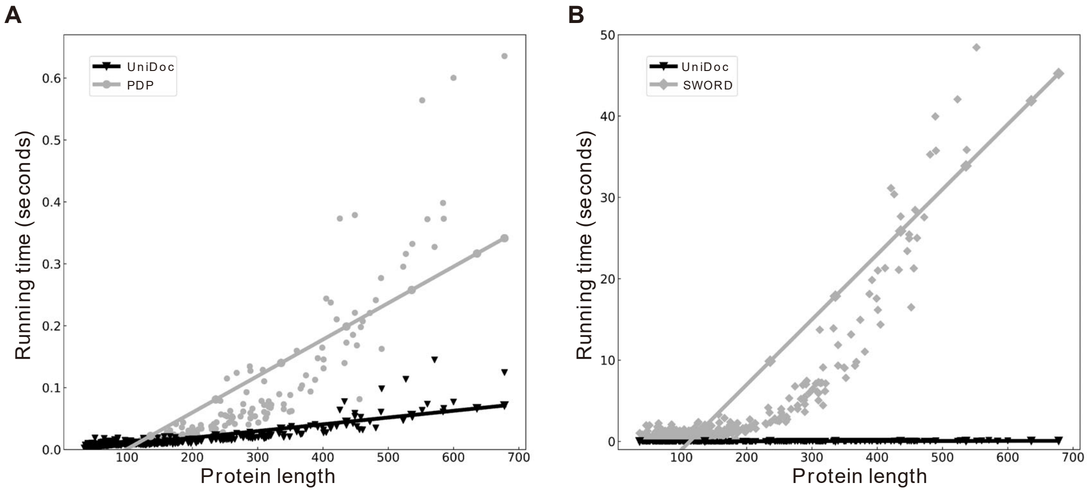

Fig. S5. Running time of UniDoc and other methods. (A) Comparison between UniDoc and PDP. (B) Comparison between UniDoc and SWORD. All methods were run locally in our cluster system. The lines were obtained based on the linear fit between the running time and the protein length.

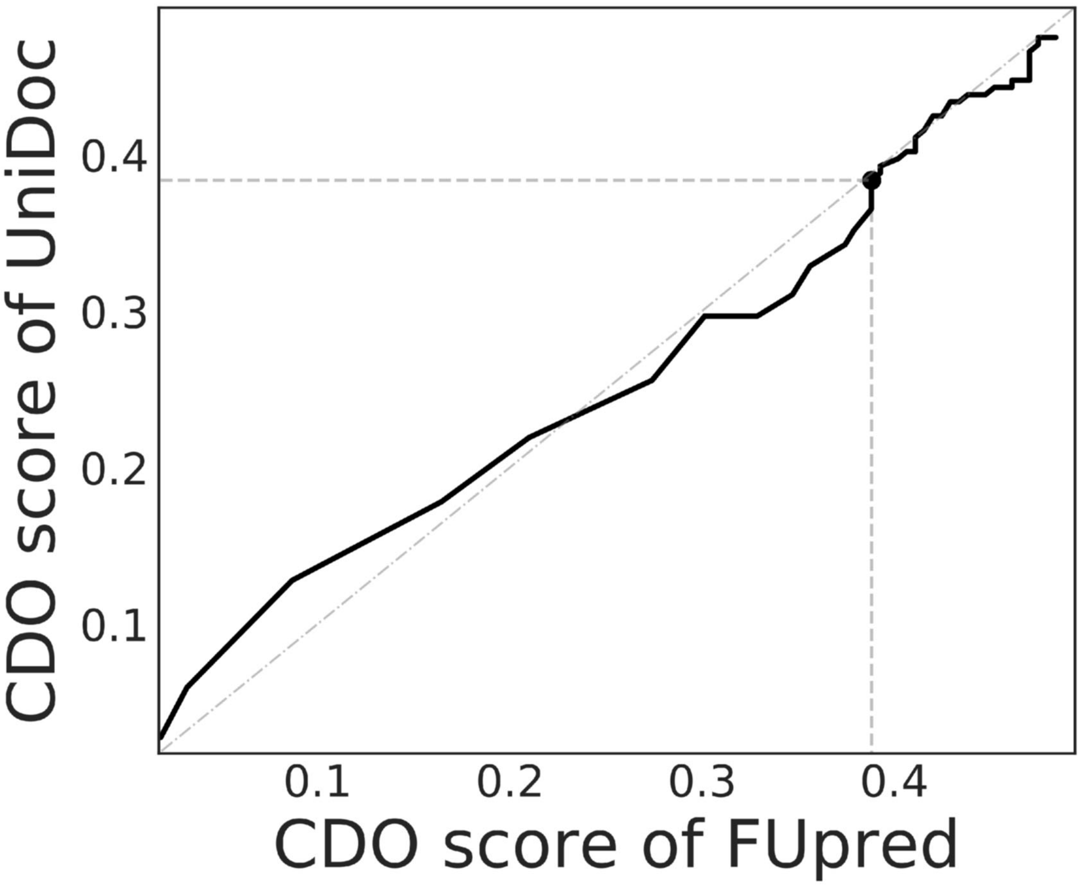

Fig. S6. Comparison with FUpred on *x*-domains targets (x>2) from the FUpred_seq dataset.

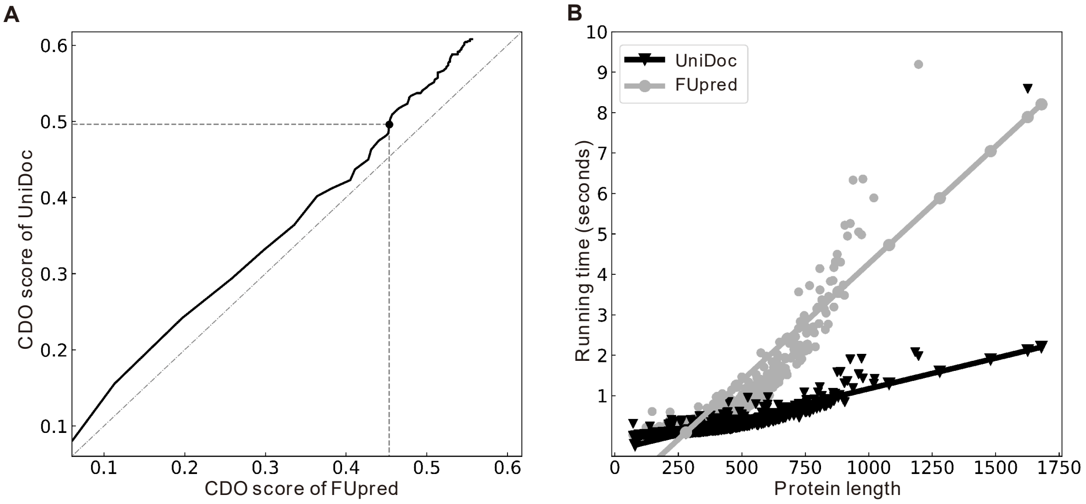

Fig. S7. Performance of sequence-based domain boundary prediction. (A) Comparison between UniDoc and FUpred based on CDO scores at different overlap thresholds on the dataset FUpred_seq. (B) Running time of FUpred based on CDO score on the dataset FUpred_seq. The lines were obtained based on the linear fit between the running time and the protein length.

Table S1. Summary of the benchmark datasets. S: SCOP, C: CATH, E: ECOD, M: Islam2363.

Dataset	#Single	#2-domain	#3-domain	#>3-domain	Discontinues-domain	Definition	Type
Islam90	68	10	7	2	1	M	3D
Broad-consensus	273	38	15	2	9	S+C+E+M	3D
Consensus	2841	567	93	22	95	S+C+E	3D
Weak-consensus	2231	428	83	27	117	S+C	3D
FUpred_seq	1700	630	155	64	133	S	1D

Metrics for assessing single-and multi-domain classification

TP(TN) represents the number of proteins that can be correctly predicted as multi-domain (single-domain), FP is the number of single-domain proteins which are incorrectly decomposed into multi-domain, and FN is the number of multi-domain proteins that were recognized as single-domain proteins.

$$\text{Pre}_m = \frac{TP}{TP+FP}, \ \text{Rec}_m = \frac{TP}{TP+FN}$$

$$\text{Pre}_s = \frac{TN}{TN+FN}, \ \text{Rec}_s = \frac{TN}{TN+FP}$$

$$ACC = \frac{TP+TN}{TP+TN+FP+FN}$$

$$MCC = \frac{TP \times TN - FP \times FN}{\sqrt{(TP+FP)(TP+FN)(TN+FP)(TN+FN)}}$$

CDO score

For structure-based domain parsing, we evaluate the accuracy based on the Islam90 set, Broad-consensus set, Consensus set and Weak-consensus set and compare UniDoc with two widely used protein domain identifying methods, such as Protein Domain Parser and SWORD. The performances of all algorithms were evaluated in terms of domain classification and domain boundaries prediction abilities. We use the CATH and SCOP annotations as the ground truth. In addition, domain assignment is considered as correct, if the predicted domain number is consistent with ground truth and each domain overlap with these assignments by over 85%. We propose the CDO (Correct Domain Overlap) score to evaluate whether an assignment is accurate or not. A detailed explanation is shown in **Table S2**, the D_j (j=1,2,3) and d_i (i=1,2,3) are the domains decomposed by annotations and algorithms, respectively. The overlap between each D_j and d_i pair, *overlap* (D_j, d_i) is first calculated; then a normalized score o_{ij} is obtained by dividing the size of D_j (**Eq. s1**). For each D_j, we calculate the maximum value of o_{ij}. The CDO score is set to 1, if every maximum values of D_j are more than a given threshold (**Eq. s2**), else the CDO score is 0.

Table S2. The overlap matrix between two assignments

	D_1	D_2	D_3
d_1	o_{11}	o_{12}	o_{13}
d_2	o_{21}	o_{22}	o_{23}
d_3	o_{31}	o_{32}	o_{33}
Score	$\max_i \{o_{i1}\}$	$\max_i \{o_{i2}\}$	$\max_i \{o_{i3}\}$

$$\text{o}_{ij} = \frac{overlap(d_i, D_j)}{N_j} \tag{s1}$$

where N_j is the size of D_j and *overlap* (D_j, d_i) is the overlap size between domain d_i by parsed and domain D_j by annotations.

$$CDO = \begin{cases} 1, & \min_j\{\max_i\{o_{ij}\}\} > 0.85 \\ 0, & \text{else} \end{cases} \tag{s2}$$

NDO score

NDO (Normalized Domain Overlap) score (Tai, et al., 2005) is widely used for measuring the quality of the predicted domains, which applies penalties to the domains if they are too small or too large. The association between the predicted domains and the answer is defined based on the maximum overlap. The penalties for the errors in predicting the number of domains are implicit in the penalties related to the extra or the missing domain boundaries. The detailed calculation steps are summarized below.

For N domains in the ground truth and M domains in the prediction, calculate the overlap o_{ij} for domain D_i $(i=1,2,\ldots,N)$ and domain D_j $(j=1,2,\ldots,M)$, and generate an N×M matrix, denoted as O. Based on the matrix O, the net overlap score is calculated. As shown in **Table S3**, the net overlap score c_j of domain D_j $(j=1,2)$ is calculated by subtracting all the remaining values from the largest value in the j-th column of O. Similarly, the net overlap score r_i of domain d_i $(i=1,2,3)$ is calculated by subtracting all the remaining values from the largest value in the i-th row of O. Finally, the net overlap scores are summed up and divided by the length of the protein to obtain the NDO score.

Table S3. The overlap matrix for the NDO score calculation.

	D_1	D_2	Net overlap score
d_1	o_{11}	o_{12}	r_1
d_2	o_{21}	o_{22}	r_2
d_3	o_{31}	o_{32}	r_3
Net overlap score	c_1	c_2	NDO score

Table S4. Single- and multi-domain classification results. 'Pre', 'Rec', 'ACC' and 'MCC' are the precision, recall, accuracy, and Matthew's correlation coefficient, respectively.

Benchmark	Method	Single		Multi		All	
		Pre	Rec	Pre	Rec	ACC	MCC
Islam90	PDP	0.967	0.853	0.630	0.895	0.862	0.668
	SWORD	0.952	0.882	0.667	0.842	0.874	0.670
	UniDoc	0.967	0.868	0.654	0.895	0.874	0.688
Broad-consensus	PDP	0.985	0.945	0.772	0.927	0.942	0.812
	SWORD	0.974	0.974	0.873	0.873	0.957	0.847
	UniDoc	0.959	0.949	0.759	0.8	0.923	0.733
Consensus	PDP	0.980	0.886	0.660	0.925	0.893	0.720
	SWORD	0.955	0.934	0.749	0.820	0.912	0.728
	UniDoc	0.957	0.932	0.745	0.824	0.911	0.729
Weak-consensus	PDP	0.983	0.870	0.636	0.937	0.883	0.710
	SWORD	0.957	0.922	0.720	0.827	0.904	0.713
	UniDoc	0.968	0.912	0.705	0.875	0.905	0.730

Table S5. Comparison between UniDoc and FUpred. The FUpred_seq dataset is used in this test.

Method	MCC	CDO	NDO
FUpred	0.784	45.35	0.804
UniDoc	**0.804**	**49.59**	**0.812**

References

Tai, C.H., *et al.* Evaluation of domain prediction in CASP6. *Proteins* 2005;61 Suppl 7:183-192.
